# Breast Implant Illness: Symptoms, Outcomes with Explantation and Potential Etiologies—A Systematic Review and Meta-analysis

**DOI:** 10.1007/s00266-025-05142-x

**Published:** 2025-08-11

**Authors:** Sofia Ferreira, António S. Barros, Marisa Marques

**Affiliations:** 1 Faculty of Medicine, University of Porto, Porto, Portugal; 2 RISE-Health, Department of Surgery and Physiology, Faculty of Medicine, University of Porto, Porto, Portugal; 3 Department of Surgery and Physiology, Faculty of Medicine, University of Porto, Porto, Portugal

**Keywords:** Breast implant illness, Explantation, Systemic symptoms, Autoimmune disease, Microbial colonization, Capsular inflammation

## Abstract

**Background:**

Breast Implant Illness (BII) is a controversial condition characterized by a broad spectrum of systemic symptoms reported by patients with breast implants, leading to an increase in explantation procedures. Its mechanisms remain unclear, with hypotheses including immune responses, microbial colonization, and psychological factors. This study analyzes potential causes, common symptoms, and the impact of explantation on symptom resolution.

**Methods:**

A systematic review following PRISMA guidelines was conducted using PubMed, Web of Science, and Scopus databases. Studies on BII symptoms and outcomes were screened based on predefined criteria. Data on demographics, implant characteristics, symptoms, explantation outcomes and potencial etiologies were extracted. Meta-analyses were performed on symptom reduction, fibromyalgia and antinuclear antibodies (ANA) positivity prevalence.

**Results:**

From 4612 identified articles, 33 met the inclusion criteria, encompassing 6048 women with an average age of 46.0 years. Symptoms appeared 6.4 years post-implantation, with explantation after 12.3 years. 81.9% of patients reported symptom improvement post-explantation, with fatigue (58.3%), joint pain (51%), and muscle pain (44%) being the most common symptoms. The prevalence of psychiatric illness, autoimmune conditions and fibromyalgia was 16.5%, 20.7% and 12%, respectively. Microbial analysis was positive on 35.2% of BII patients. ANA positivity prevalence was estimated at 24% and capsular inflammation at 58.4%. Implant rupture and capsular contracture rates were 21.4% and 44.4%, respectively.

**Conclusion:**

This review supports BII as a real, multifactorial clinical entity involving immune dysregulation, chronic inflammation, and microbial biofilms. These findings underscore the importance of individualized assessment, screening for autoimmune and psychiatric conditions, informed consent and adherence to surgical protocols such as the 14-Point Plan and antimicrobial irrigation to reduce complications.

**Level of Evidence III:**

This journal requires that authors assign a level of evidence to each article. For a full description of these Evidence-Based Medicine ratings, please refer to the Table of Contents or the online Instructions to Authors www.springer.com/00266.

**Supplementary Information:**

The online version contains supplementary material available at 10.1007/s00266-025-05142-x.

## Introduction

Breast implant illness (BII) is a controversial and poorly defined condition, characterized by a broad spectrum of systemic symptoms self-reported by patients with breast implants [1, 2]. Since their introduction in the 1960s, silicone breast implants have been hypothesized to be associated with systemic disease [3–5]. While breast augmentation remains one of the most commonly performed cosmetic procedures worldwide [6], an increasing number of patients are seeking explantation due to concerns over BII, with explantation procedures rising significantly in recent years [7].

Some patients report symptom improvement following explantation [8], yet the lack of controlled studies makes it difficult to determine whether explantation is a definitive treatment [9, 10].

More than 100 symptoms have been attributed to BII, with no specific configuration, making it difficult to establish clear diagnostic criteria [9–11], relying on symptom presentation and ruling out alternative causes [12]. These systemic symptoms can be subdivided into three categories: neurological/musculoskeletal, immunological and vascular manifestations [13].

Shoenfeld et al*.* proposed a set of major and minor diagnostic criteria for ASIA (Autoimmune Syndrome Induced by Adjuvants), with the major being the exposure to an adjuvant prior to clinical manifestations, the appearance of typical clinical manifestations (myalgia, fatigue, arthralgia, poor sleep, neurologic manifestations, cognitive impairment or pyrexia/dry mouth). Also, the explantation inducing improvement and typical biopsy of involved organs. As minor criteria, the appearance of autoantibodies or antibodies directed at the adjuvant, other clinical manifestations, specific HLA (HLA DRB1 or HLA DBQ1) and the evolvement of an autoimmune disease [14].

Some researchers suggest that symptoms arise due to an immune response triggered by silicone acting as an adjuvant [15], while others hypothesize the involvement of bacterial biofilms [16], heavy metals [25] or BII as a somatization disorder [17]. However, no definitive causative link has been established between breast implants and systemic disease, and most patients have normal physical examinations and laboratory findings [10, 16]. Its differentiation from conditions with overlapping symptoms, such as fibromyalgia, remains challenging [9, 12].

Given the growing number of patients reporting symptoms and seeking explantation, along with the lack of consensus on the etiology and diagnostic criteria of BII, a comprehensive evaluation of its potential mechanisms is essential.

The aim of this systematic review is to comprehensively evaluate the potential mechanisms underlying Breast Implant Illness (BII) by analyzing the interplay between immunological, microbial, inflammatory, psychological, and environmental factors that may contribute to the development and persistence of symptoms. Furthermore, this study aims to identify the most common symptoms described by these patients and explore the effects of explantation on symptom resolution or improvement.

By synthesizing existing literature and emerging evidence, our goal is to provide a clearer understanding of BII’s pathophysiology, contributing to more informed clinical decision-making and patient care.

## Methods

This systematic review followed the Preferred Reporting Items for Systematic Reviews and Meta-Analyses (PRISMA).

### Study Selection

The literature searches were performed using the PubMed, Web of Science, and Scopus electronic databases from inception to November 2024.

The query “(breast implant illness) OR (BII) OR (breast implant syndrome) OR (silicone implant syndrome) OR (silicone implant illness) OR (silicone implant incompatibility syndrome) OR (ASIA syndrome) OR (autoimmune syndrome induced by adjuvants) AND (symptoms) OR (diagnosis) OR (management) OR (treatment) OR (long term outcomes) OR (follow-up) OR (adverse effects) OR (prognosis)” was used for the PubMed database search.

The query ‘‘breast implant illness (All Fields) or BII (All Fields) or breast implant syndrome (All Fields) or silicone implant syndrome (All Fields) or silicone implant illness (All Fields) or silicone implant incompatibility syndrome (All Fields) or ASIA syndrome (All Fields) or autoimmune syndrome induced by adjuvants (All Fields) and symptoms (All Fields) or diagnosis (All Fields) or management (All Fields) or treatment (All Fields) or long term outcomes (All Fields) or follow-up (All Fields) or adverse effects (All Fields) or prognosis (All Fields)” was used for the Web of Science database search.

The query ‘‘TITLE-ABS-KEY (breast implant illness) OR TITLE-ABS-KEY (BII) OR TITLE-ABS-KEY (breast implant syndrome) OR TITLE-ABS-KEY (silicone implant syndrome) OR TITLE-ABS-KEY (silicone implant illness) OR TITLE-ABS-KEY (silicone implant incompatibility syndrome) OR TITLE-ABS-KEY (ASIA syndrome) OR TITLE-ABS-KEY (autoimmune syndrome induced by adjuvants) AND TITLE-ABS-KEY (symptoms) OR TITLE-ABS-KEY (diagnosis) OR TITLE-ABS-KEY (management) OR TITLE-ABS-KEY (treatment) OR TITLE-ABS-KEY (long term outcomes) OR TITLE-ABS-KEY (follow-up) OR TITLE-ABS-KEY (adverse effects) OR TITLE-ABS-KEY (prognosis)” was used for the Scopus database search.

For the eligibility assessment, two investigators selected relevant studies by title and abstract at first. Then, the full text article screening was carried out by both investigators separately. Relevant studies were identified based on predefined inclusion and exclusion criteria to ensure the selection of high-quality and pertinent studies.

### Inclusion and Exclusion Criteria

The selection process was guided by specific inclusion criteria: primary studies, such as randomized controlled trials, prospective or retrospective studies, cohort, cross sectional or case series that focused on the Breast Implant Illness, with reports of symptoms, outcomes with explantation or investigation of a possible etiology.

Exclusion criteria included review articles, case reports, animal or cadaver studies, the use of Poly Implant Prothese (PIP), studies in which the population was not exclusively cisgender women and studies focused on implants in areas other than the breast. The search also excluded studies that could not be accessed in full text, with overlapping data or no full discrimination of symptoms or outcomes.

### Quality Assessment

The risk of bias was calculated using the National Institute of Health Quality Assessment tools.

For most studies, the Quality Assessment Tool for Observational Cohort and Cross- Sectional Studies was applied, in which the studies were classified as Poor if scored 1-4, Fair if scored 6–10 and Good if scored 11–14. For four studies [10, 18–20], the Quality Assessment Tool for Case Series Studies was applied, in which the studies were classified as Poor if scored 1–3, Fair if scored 4–6 and Good if scored 7–9. For one study [21] the Quality Assessment Tool for Before-After (Pre-Post) Studies With No Control Group was applied and for three studies [22–24] the Quality Assessment of Case-Control Studies was applied, in which the studies were classified as Poor if scored 1–4, Fair if scored 5–8 and Good if scored 9–12. The results can be viewed in Supplemental Table 1.

### Data Collection

Data collection was performed independently from the reports by one reviewer and then revised by the other two authors. It included year of publication, study design, number of patients, mean age, reason for implantation, type of implant, implant surface, mean follow up time, mean time until beginning of symptoms and from implantation to explantation.

Regarding the outcomes, the revised data encompassed number of patients that reported improvement of symptoms after explantation, number of patients that reported each symptom and mean number of symptoms pre and post explant.

In order to establish possible etiologies, data included the number of patients with previous history of psychiatric illness, autoimmune diagnosis, fibromyalgia, positive microbial culture, implant rupture or bleed, capsular contracture, capsular inflammation and positive antinuclear antibodies (ANA).

### Synthesis of Results

A meta-analysis regarding symptom reduction following explantation was performed. The data analyzed were continuous variables in the form of mean differences and standard deviations. Another two meta-analysis were performed for the Prevalence of Fibromyalgia and ANA positivity in BII Patients. The data analyzed were categorical variables in the form of proportions and confidence intervals (IC 95%).

The results were analyzed in a random effects model. Heterogeneity was measured via the I^2^ test. For all analyses *p* < 0.05 was considered significant.

## Results

**Fig. 1:**
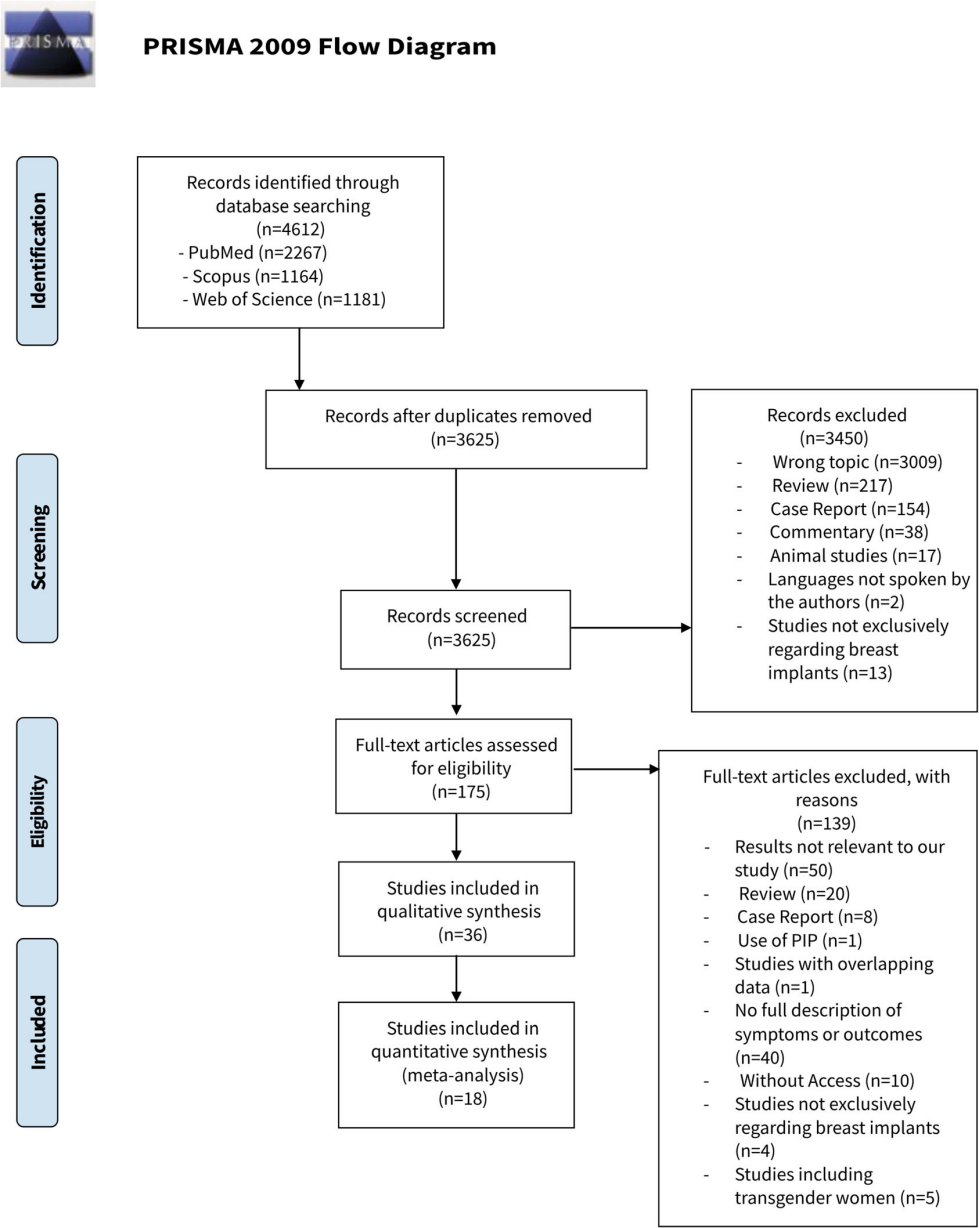
Flowchart according to PRISMA guidelines

A total of 4612 articles were initially identified through database searches. After eliminating duplicates using Rayyan systematic review management platform (Rayyan Systems, Cambridge, Massachusetts, United States), 3625 articles underwent screening based on their titles and abstracts. Following this, 175 full-text articles were reviewed for eligibility, and 36 studies met the criteria for inclusion in this systematic review [1, 3–5, 9–11, 15, 16, 18–44]. Among these, four [11, 25, 39, 40] were considered as a single study, as they represented different parts of the same research paper divided into four sections. The selection process is visually summarized in a PRISMA flowchart in Fig. 1.

The dataset encompasses 33 distinct studies investigating Breast Implant Illness, collectively including 6048 patients. Most studies (> 60%) were published in the last decade (2014–2024). Only 7 studies were prospective [1, 11, 15, 27, 29, 34, 35].

The mean age was 46.0 years (range: 39.2–54.0 years). The 17 studies reporting follow-up duration had a mean follow-up of 16.3 months. The characteristics of the papers are detailed in Supplemental Table 2.

**Fig. 2:**
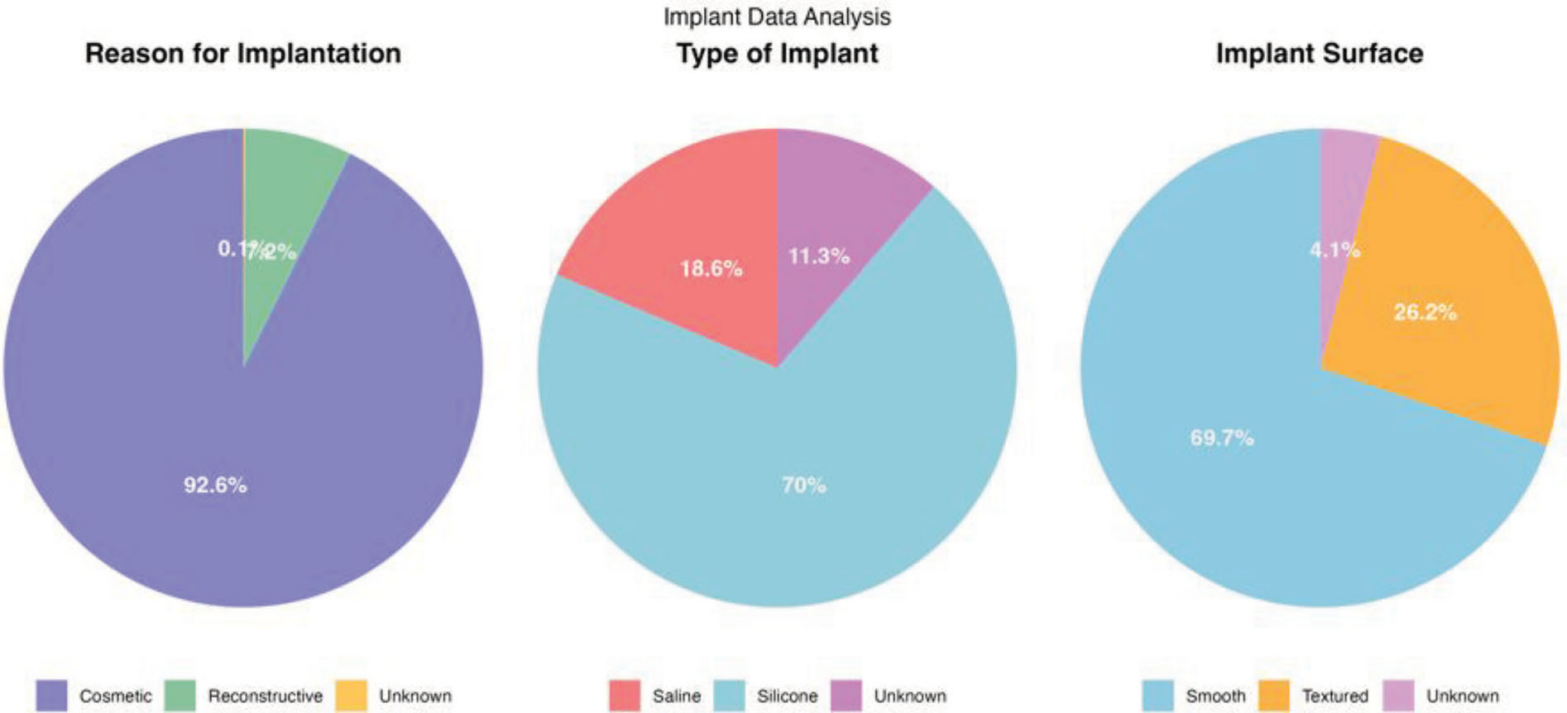
Pie charts that represent reason for implantation, type of implant and implant surface for the analyzed papers

Cosmetic enhancement was the primary reason for implantation (92.6%). Silicone implants were substantially more common (70%) than saline implants (18.6%). Similarly, smooth-surfaced implants were preffered in most cases (69.7%) compared to textured implants (26.2%). This information can also be found in Fig. 2.

**Fig. 3:**
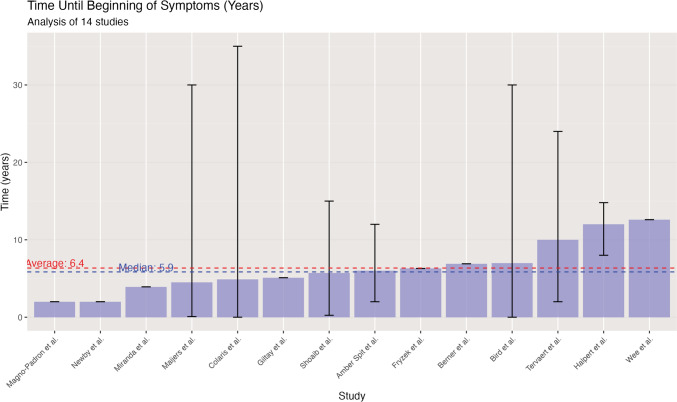
Average time (in years) from breast implantation to the onset of systemic symptoms, as reported across 14 clinical studies included in this systematic review. The vertical bars indicate the mean time to symptom onset (in years) for each study, with error bars representing the reported minimum and maximum values. Studies are arranged from left to right in ascending order of mean time to symptom onset

**Fig. 4:**
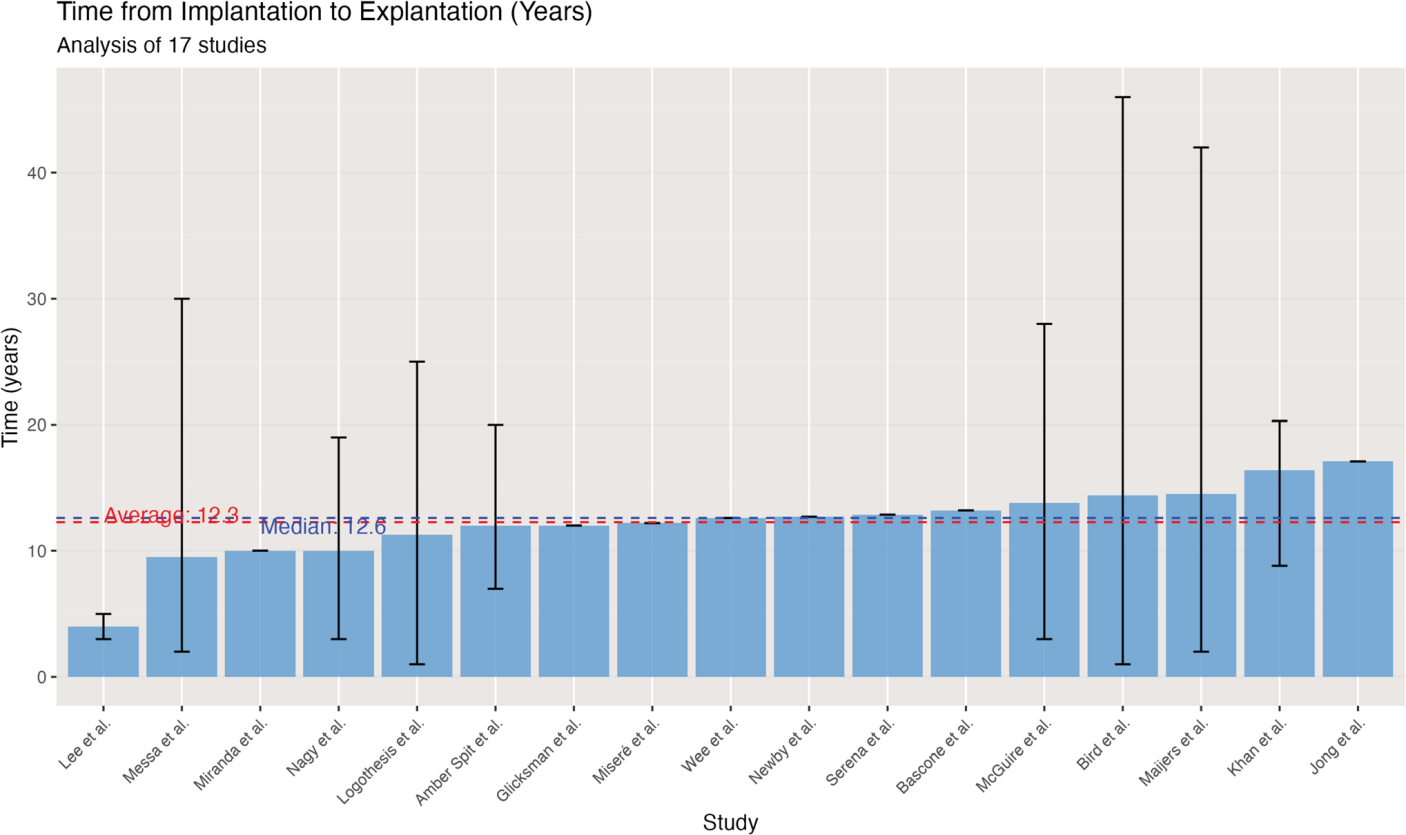
Time from breast implantation to explantation across 17 clinical studies

Regarding the timing of symptom onset, on average, symptoms began to appear 6.4 years after implantation, with a median value of 5.9 years, as seen in Fig. 3. The average duration from implantation to explantation was 12.3 years, with the median at 12.6 years. This symmetry between mean and median suggests a relatively normal distribution of reported times across studies, with more than half of all studies (52.9%) reporting average implant durations between 10 and 13 years. These results are presented in Fig. 4.

**Fig. 5:**
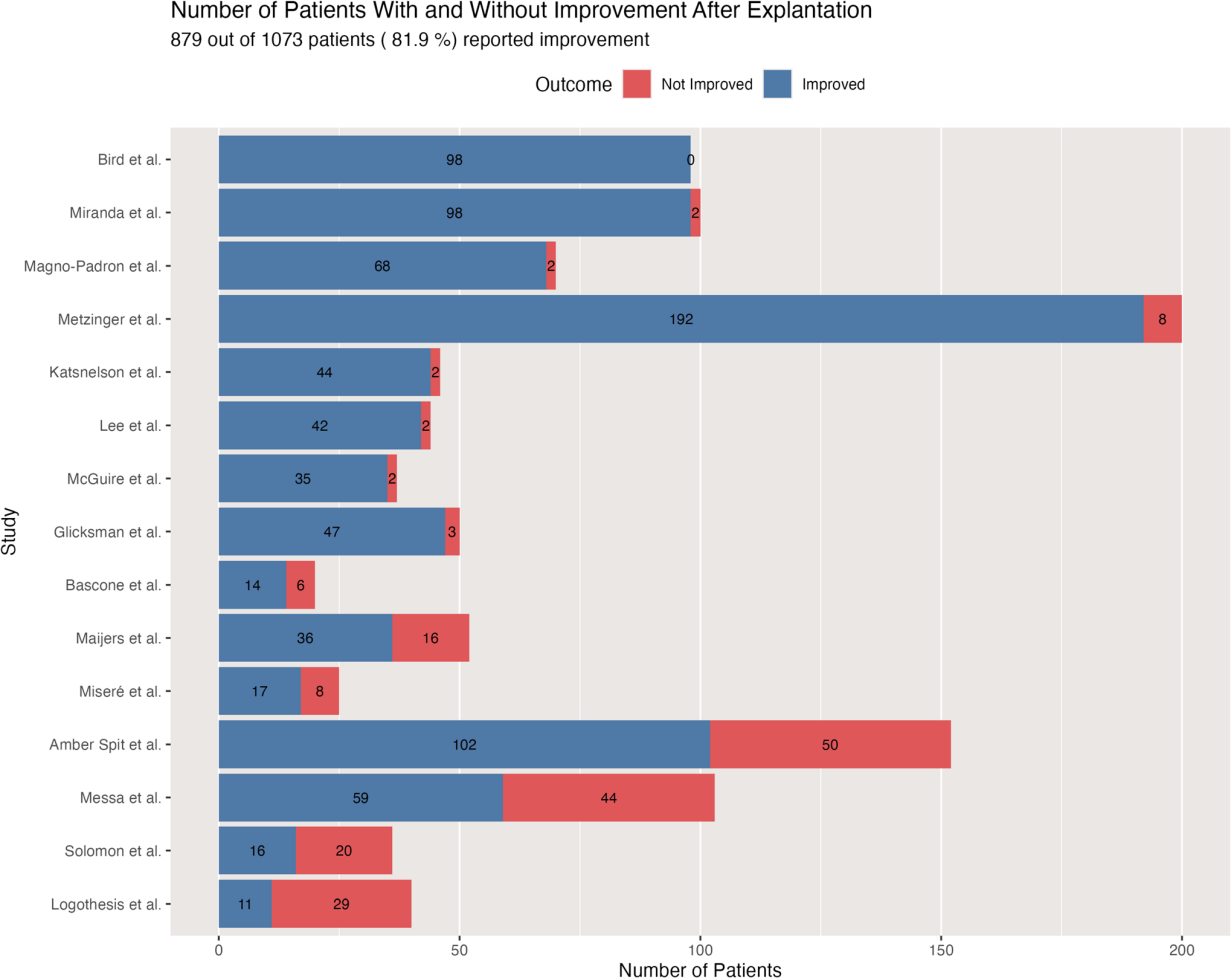
Patient outcomes after Breast Implant explantation: improved vs. non-improved cases across 15 clinical studies

As shown in Fig. 5, the overall weighted average improvement of symptoms after explantation rate was 81.9% across all studies, ranging from 100% [29] to 27.5% [30]. Eight studies [1, 4, 5, 10, 11, 16, 18, 29] reported improvement rates exceeding 90%, while only two studies [30, 36] showed improvement rates below 50%.

**Fig. 6:**
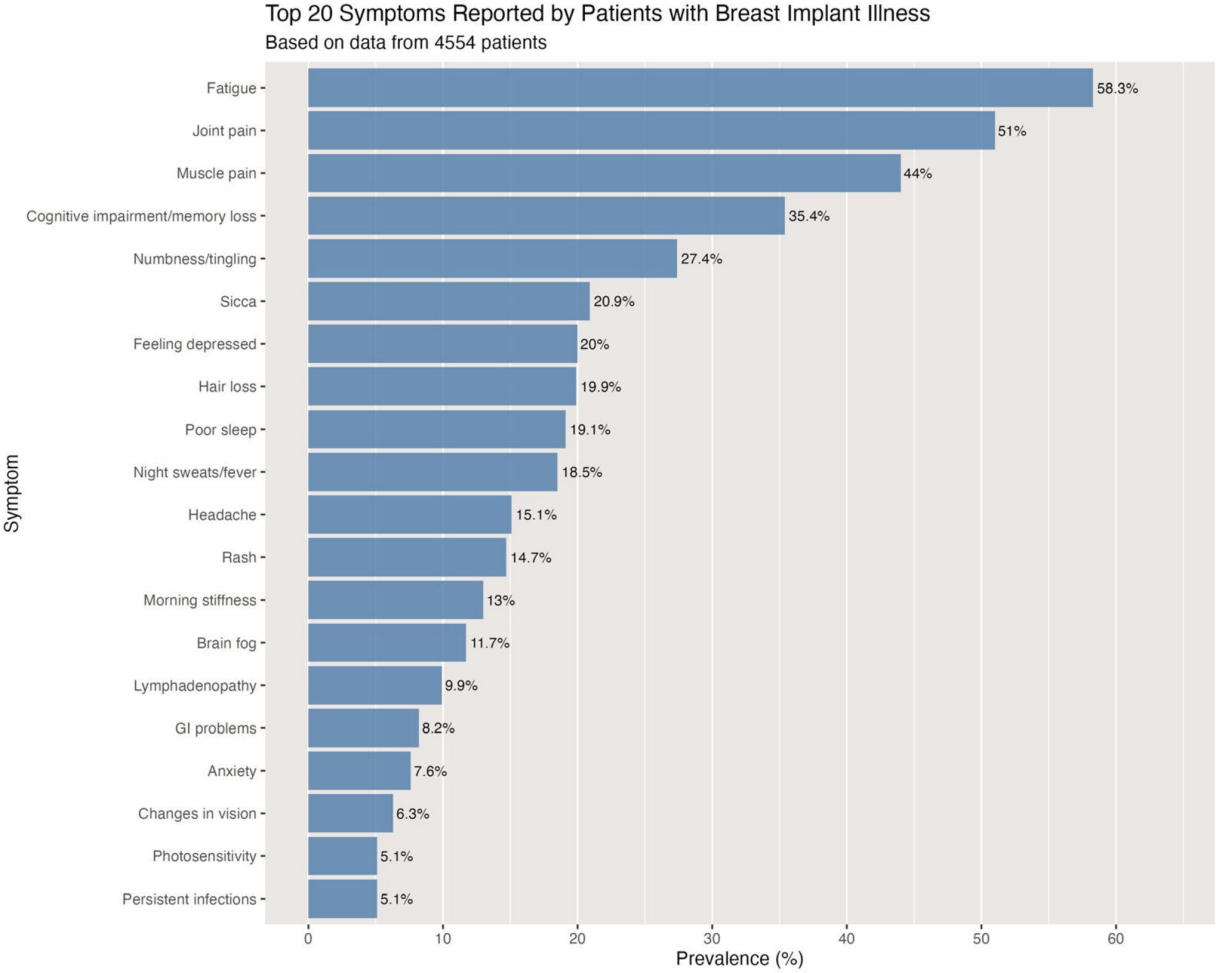
The 20 most prevalent symptoms in Breast Implant Illness

**Fig. 7:**
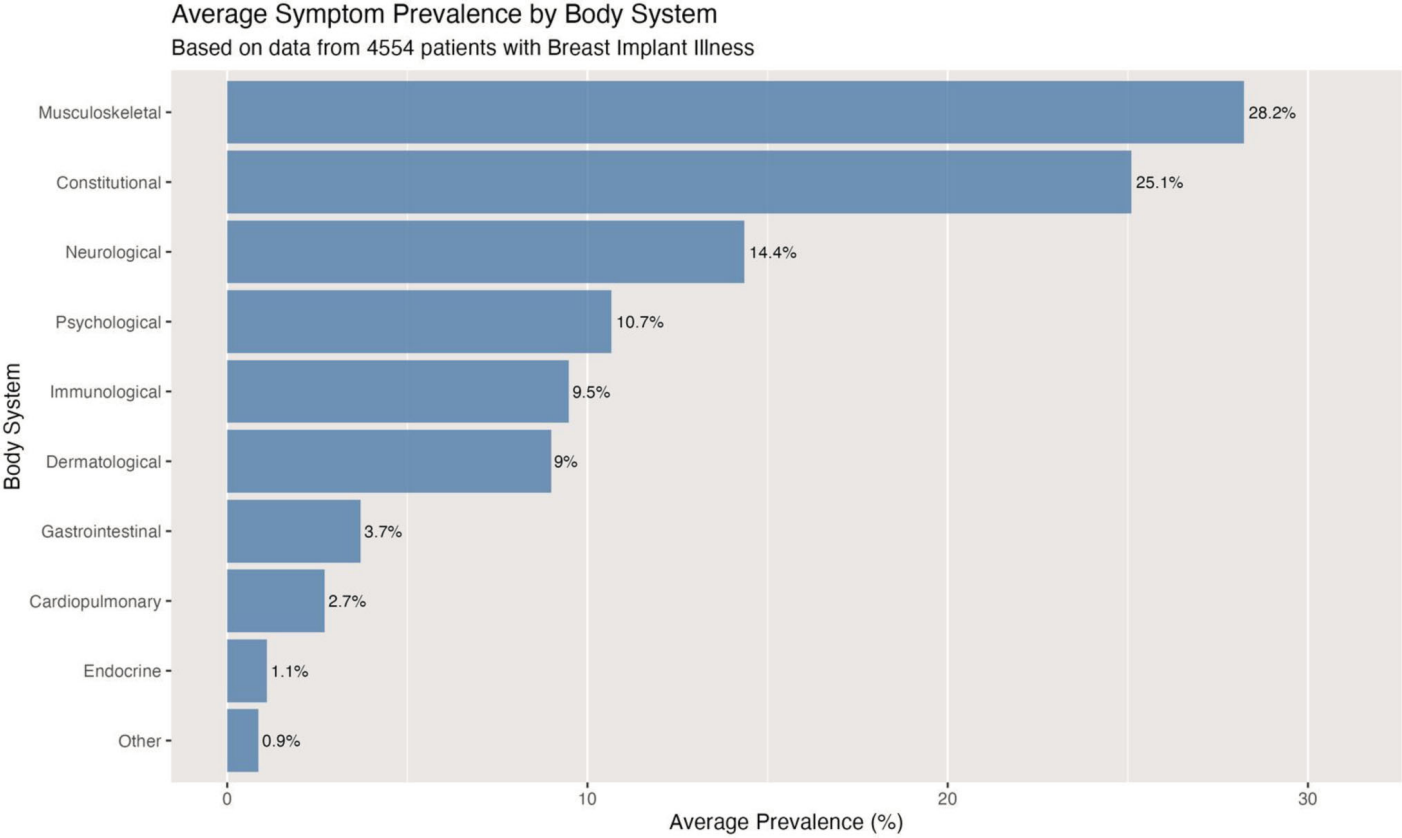
Average symptom prevalence by body system among 4554 patients with Breast Implant Illness

**Fig. 8:**
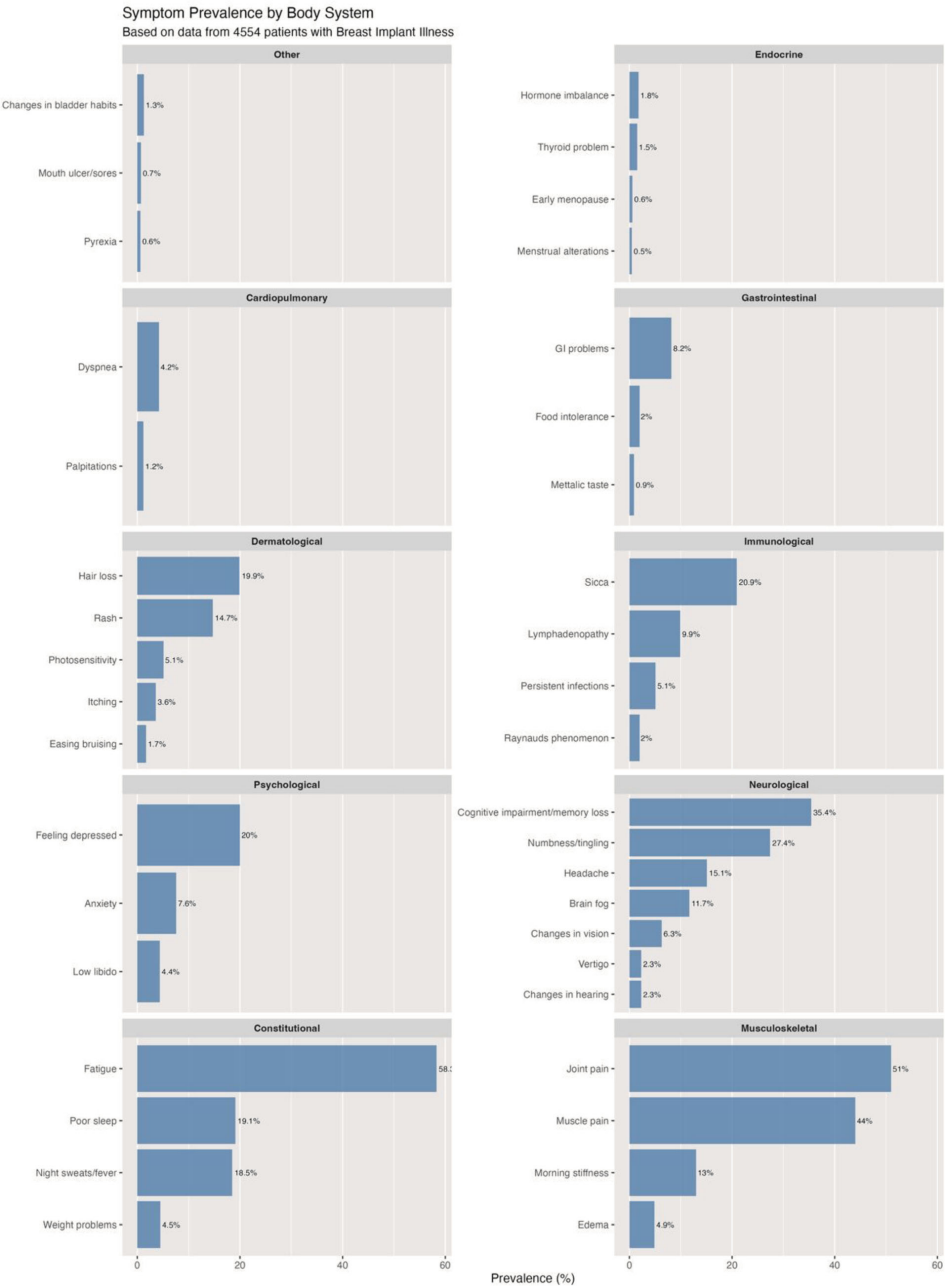
Comprehensive system-based analysis of breast implant illness: a multi-faceted symptom profile across body systems

The horizontal bar chart represented in Fig. 6 shows the top 20 symptoms reported by patients with Breast Implant Illness, based on data from 4554 patients. Fatigue is the most commonly reported symptom, affecting 58.3% of patients, followed by joint pain (51%) and muscle pain (44%). As shown in Fig. 7, musculoskeletal symptoms show the highest average prevalence at 28.2%, closely followed by constitutional (25.1%) and neurological symptoms (14.4%). Fig. 8 represents a comprehensive system-based analysis of all the symptoms described by the patients included in this systematic review.

Four studies [4, 5, 10, 39] included data regarding the symptom burden. The average number of symptoms before explantation across these studies was 12.99 (SD = 6.11). After explantation, the average symptom count decreased to 6.94 (SD = 7.38). Three of the four studies demonstrated not just statistically significant but clinically meaningful reductions in symptom burden (> 65% reduction).

**Fig. 9:**
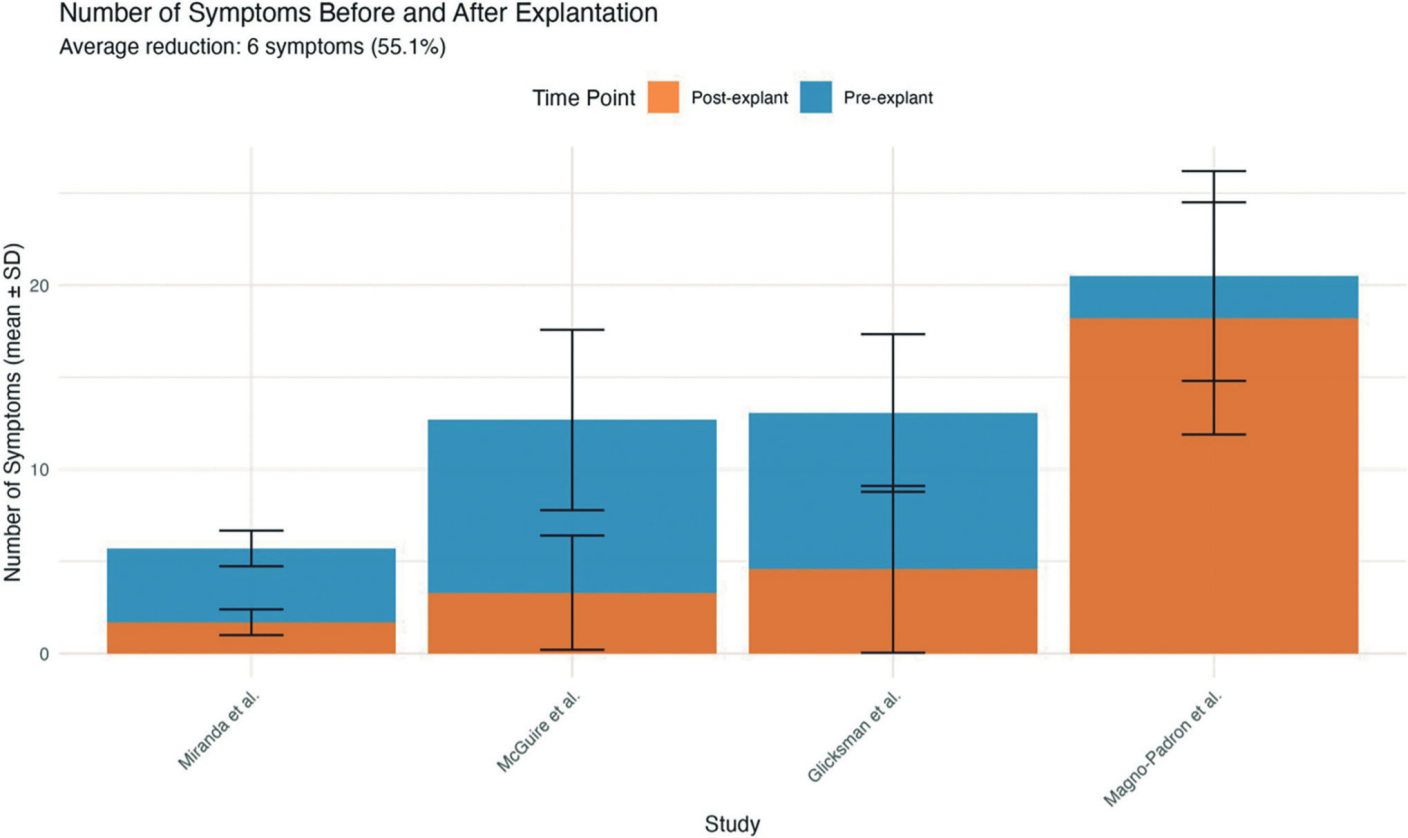
Symptom burden before and after breast implant explantation: variable responses across four clinical studies with standard deviations

As shown in Fig. 9, the average percentage reduction in symptoms following explantation is 55.1% or 6 symptoms, with considerable patient-to-patient variation in both pre- and post-explantation symptom experience.

**Fig. 10:**
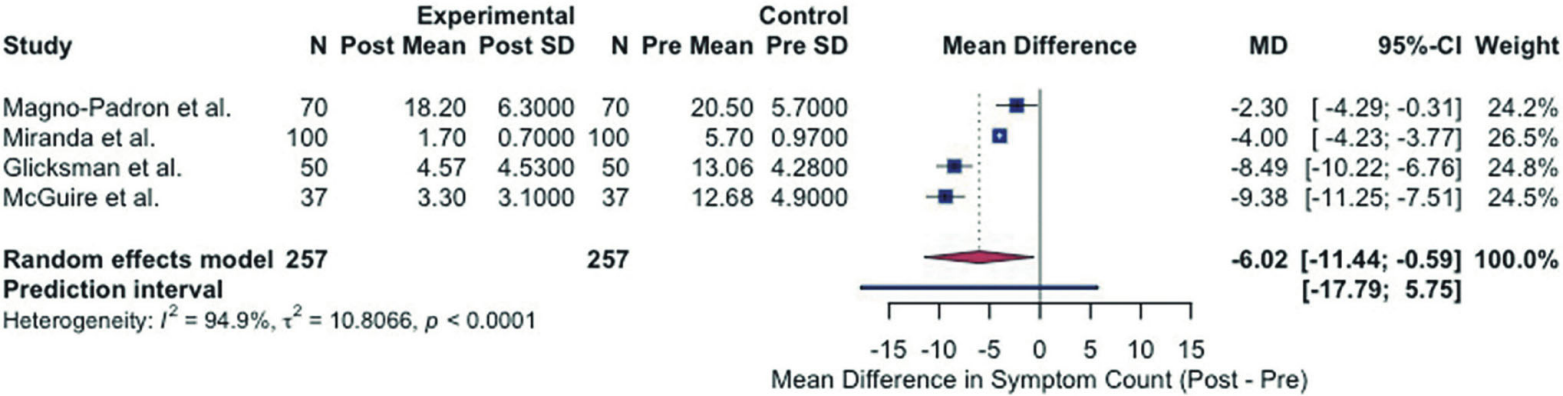
Meta-analysis: forest plot of symptom reduction following breast implant explantation

Figure 10 presents the results of a random-effects meta-analysis examining the change in symptom count following explantation across four clinical studies with a total of 257 patients. The meta-analysis reveals a statistically significant overall reduction in symptoms after explantation, with a pooled mean difference of − 6.02 symptoms (95% CI: − 11.44 to − 0.59, *p* < 0.0001). This indicates that, on average, patients experienced approximately six fewer symptoms following explantation. However, there is extreme heterogeneity among the included studies (I^2^ = 94.9%, τ^2^ = 10.81, *p *< 0.0001), suggesting substantial variability in these effects. Despite the variability, all four individual studies show a statistically significant reduction in symptoms.

**Fig. 11:**
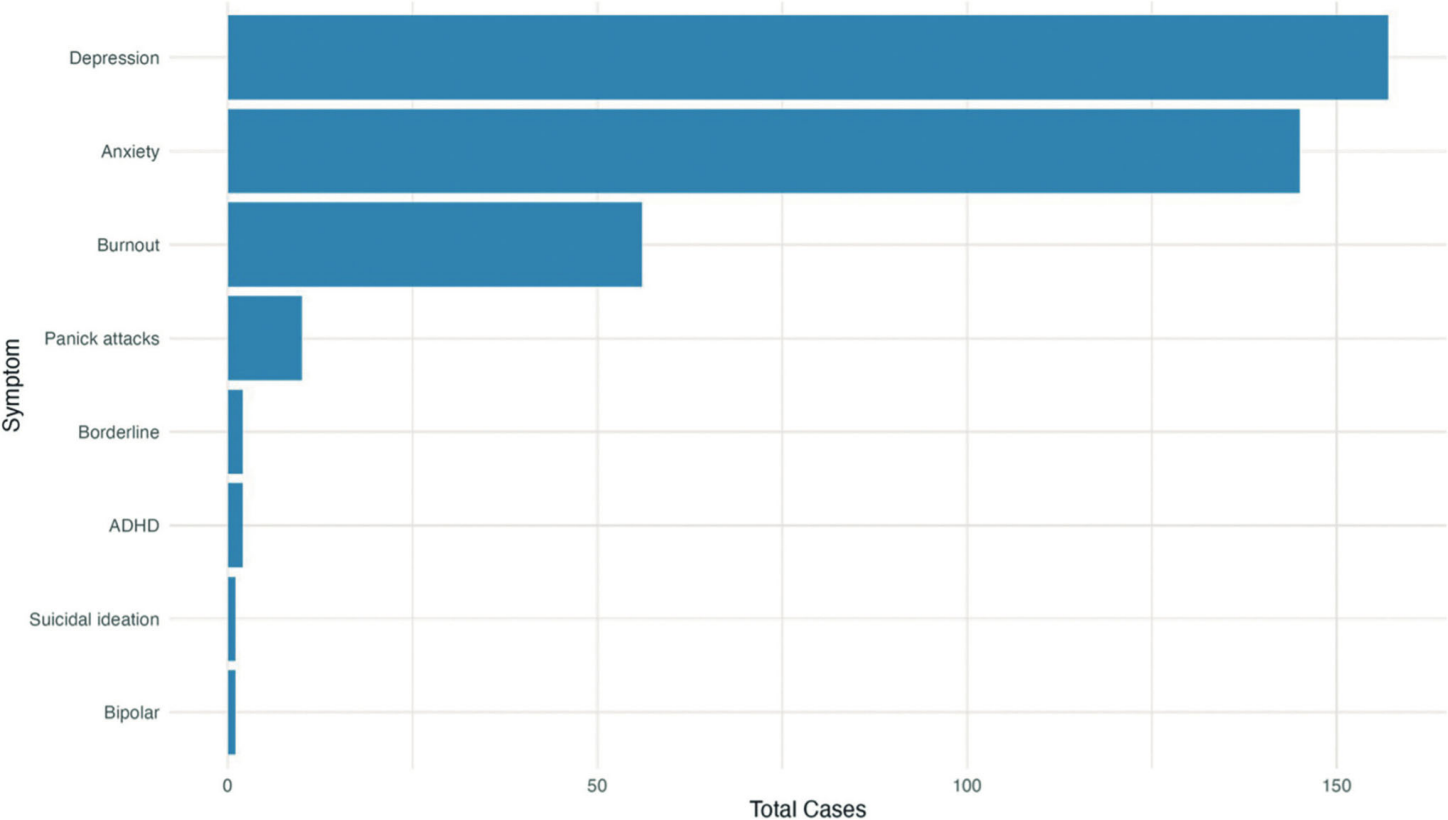
History of diagnosed psychiatric illness in breast implant illness patients: aggregated data from 10 clinical studies

Approximately 16.5% of individuals with BII (338 out of 2045 patients) had a reported history of mental illness, with an overall mean prevalance of 26.82%. The horizontal bar graph shown in Fig. 11 presents a view of the varying prevalence of different mental health diagnosis prior to implantation experienced by patients with BII. Depression and anxiety emerge as the most prominent psychiatric conditions.

**Fig. 12:**
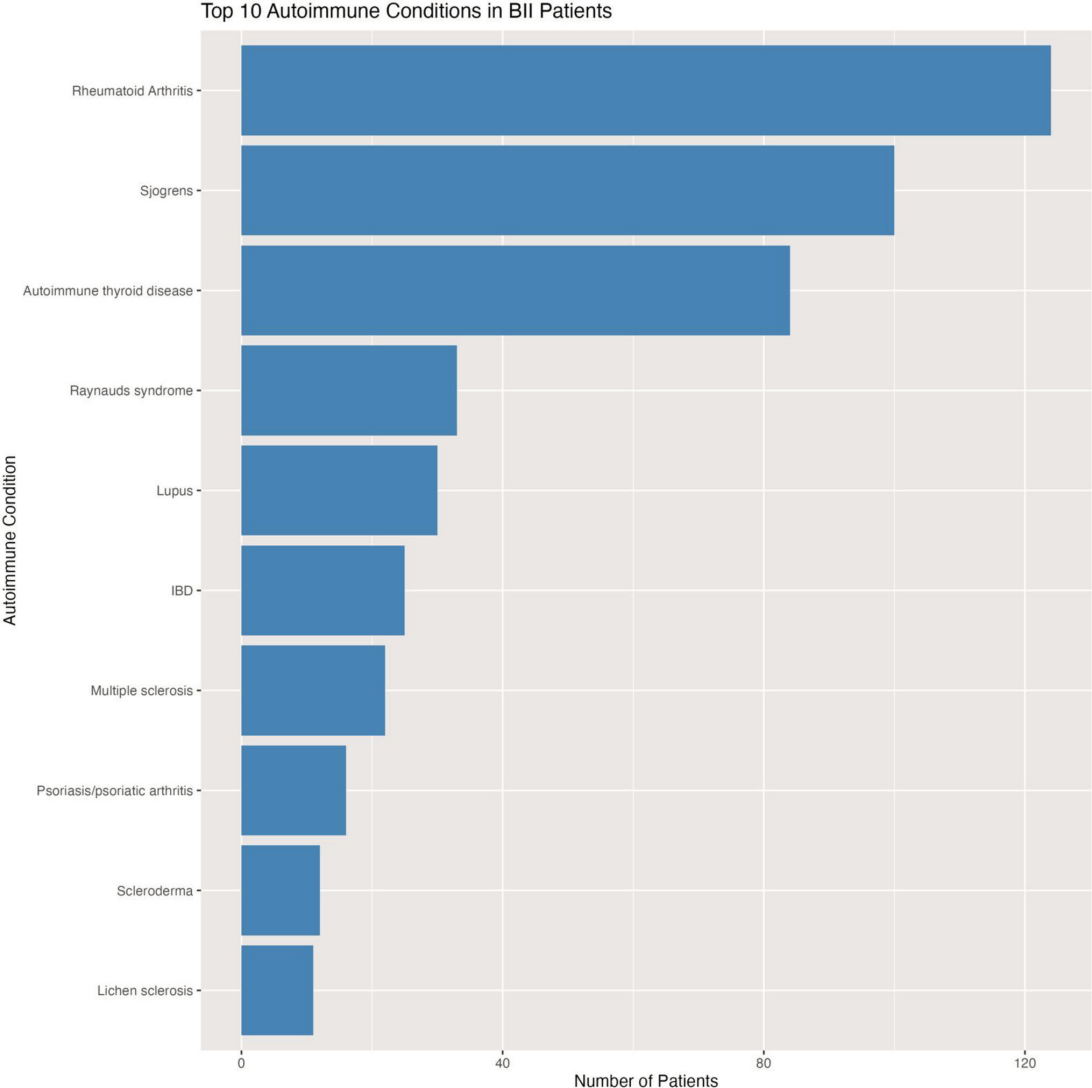
Distribution of autoimmune conditions among BII patients: aggregated data from 19 clinical studies

Approximately 20.7% of individuals with BII (680 out of 3280 patients) had a reported history of autoimmune diagnosis, with an overall mean prevalence of 28.8%. The prevalence of the top 10 autoimmune conditions identified among patients with BII prior to implantation is shown in Fig. 12. Rheumatoid Arthritis stands as the most reported autoimmune condition, with approximately 124 cases. This is followed by Sjögren’s Syndrome, with about 100 cases.

**Fig. 13:**
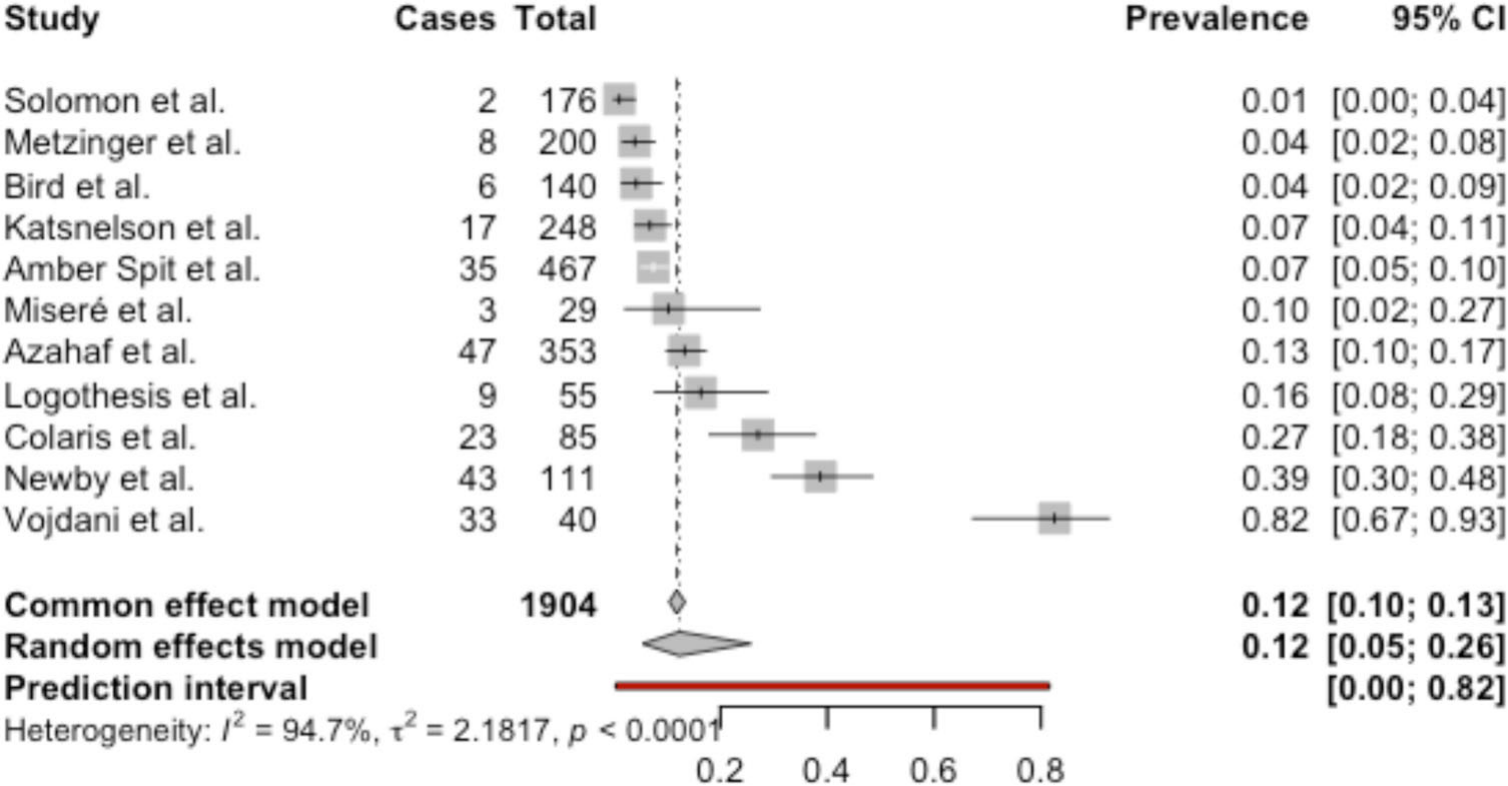
Meta-analysis: forest plot of prevalence of fibromyalgia in BII patients

The forest plot in Fig. 13 provides a meta-analysis of 11 studies regarding the prevalence of Fibromyalgia. There is a high statistical heterogeneity between studies, as indicated by the I^2^ value of 94.7% (*p* < 0.0001). The random effects model, which accounts for this heterogeneity, estimates the pooled prevalence of fibromyalgia among BII patients at 12% (95% CI: 5–26%).

**Fig. 14:**
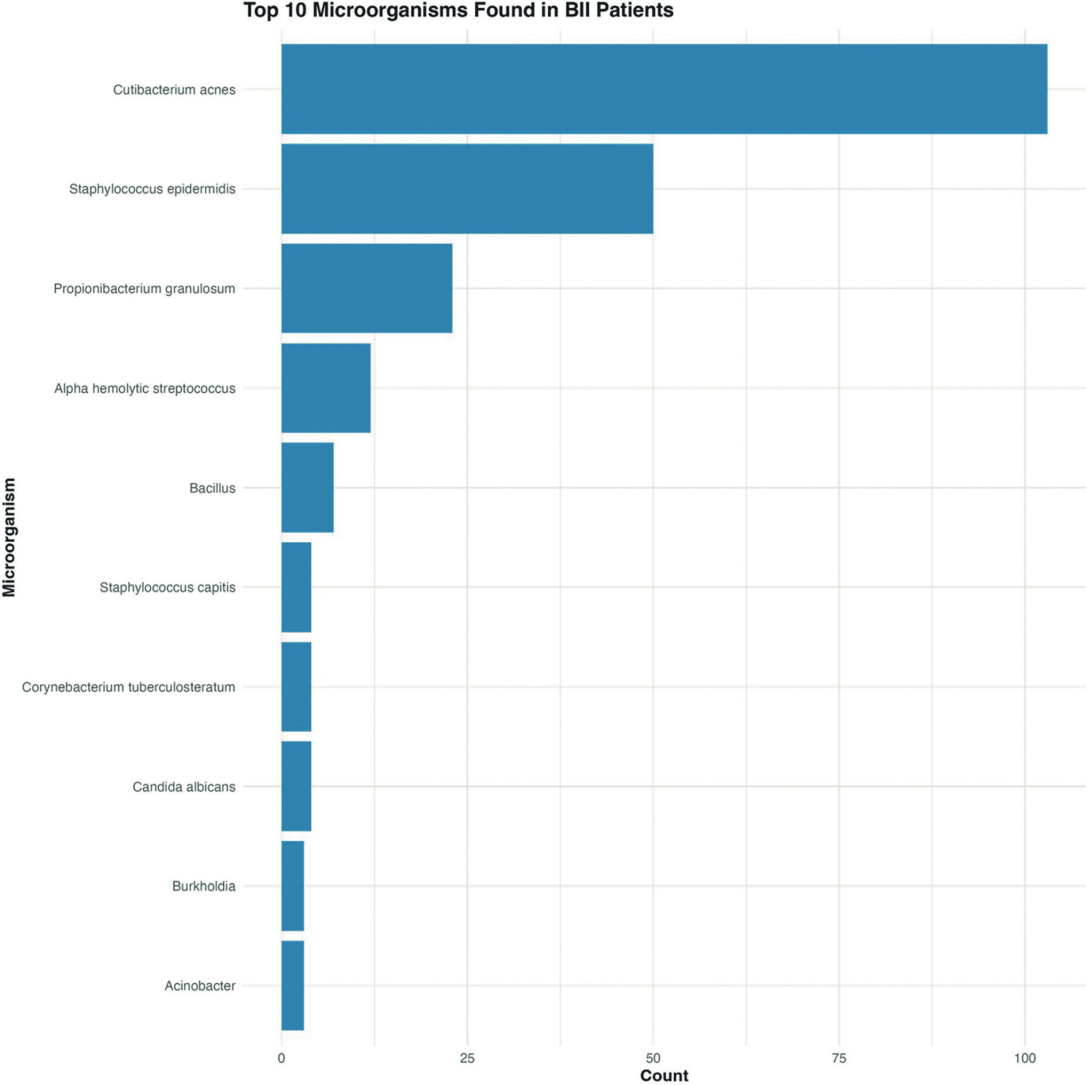
Frequency distribution of the top 10 microorganisms identified in BII patients: data aggregated from four clinical studies

Figure 14 illustrates the frequency distribution of the ten most commonly isolated microorganisms in the capsules of patients with BII across four clinical studies [1, 16, 18, 25] encompassing 548 patients.

The visualization reveals a dominance of *Cutibacterium acnes*, which was identified in 103 cases, making it more than twice as prevalent as any other microorganism. *Staphylococcus epidermidis* appears as the second most common isolate with 50 cases and *Propionibacterium granulosum* as the third (23 cases). Gram-positive bacteria dominated the microbial findings, accounting for 91.03% of all isolates. Overall, 193 out of 548 BII patients (35.22%) had positive microbial growth.

Regarding implant rupture/bleed, the overall rate was 21.4% (481 out of 2251 patients). The mean rupture rate across studies was 28.5%. About capsular contracture, the overall rate was 44.4% (1136 out of 2560 patients) with a mean rate of 40.6%.

**Fig. 15:**
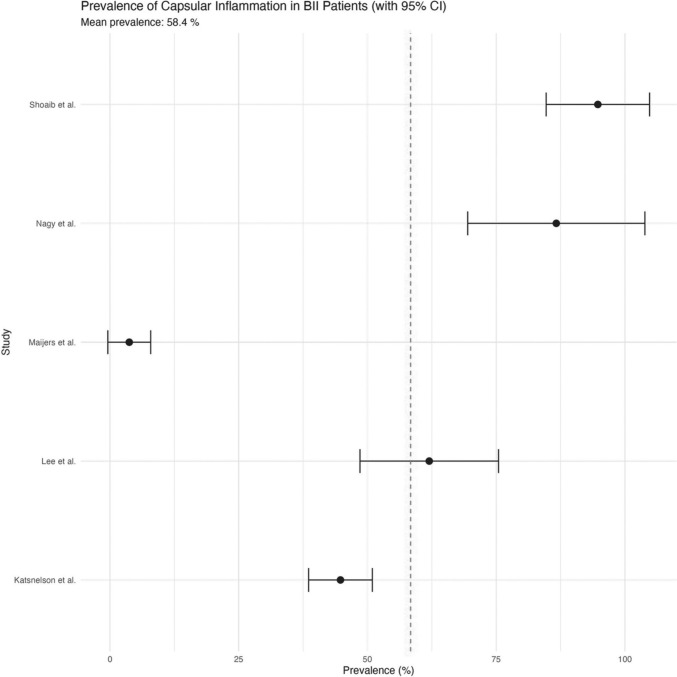
Forest plot of capsular inflammation prevalence in BII patients

The forest plot in Fig. 15 displays the prevalence of capsular inflammation among BII patients across five studies [1, 18, 24, 26, 34], with an overall mean prevalence of 58.4%. The visualization shows substancial heterogeneity in imflammation rates with the lowest being 3.8% [26] and the highest being 94.7% [34].

**Fig. 16:**
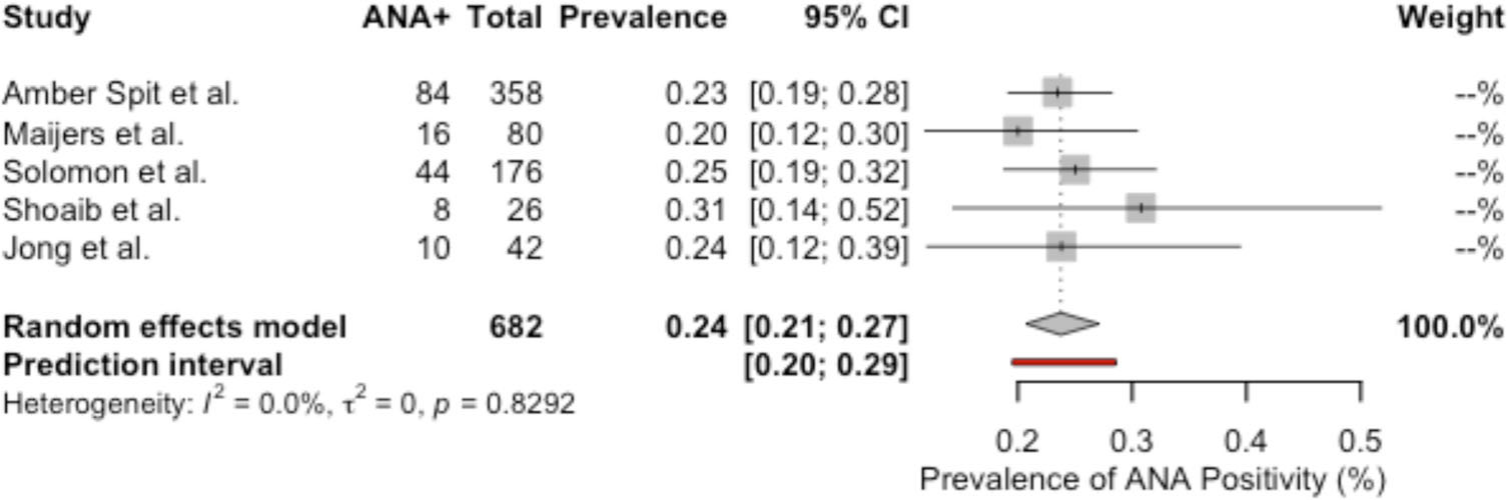
Meta-analysis: forest plot of prevalence of ANA positivity in BII patients

The forest plot in Fig. 16 provides a meta-analysis of 5 studies [15, 26, 34, 36, 44] regarding the prevalence of ANA positivity among BII patients. Statistical heterogeneity is not present, as indicated by the I^2^ value of 0.0% (*p *= 0.8292). The random effects model estimates the pooled prevalence of ANA positivity among BII patients at 24% (95% CI: 21–27%), with individual study prevalences ranging from 20% [26] to 31% [34].

## Discussion

### Symptoms

Research has identified a pattern of self-reported symptoms associated with Breast Implant Illness (BII) [26]. These are believed to result from a complex interaction between biophysiology and psychology [5].

Commonly reported symptoms include fatigue, arthralgia, myalgia, cognitive impairment, and peripheral neurological symptoms, [15] as shown in Fig. 6. Other relevant complaints encompass sleep disturbances and dry eyes and mouth [45]. This is supported by the findings of the present systematic review where the data reveals a clear pattern where musculoskeletal and constitutional symptoms dominate the clinical presentation of BII (Fig. 7), demonstrating the systemic nature of this entity, with symptoms manifesting across multiple body systems but showing clear variation in prevalence between physiological domains (Fig. 8).

### Efficacy of Explantation

Numerous studies indicate that explantation with or without capsulectomy leads to a significant reduction or resolution of symptoms [1, 2, 4, 5, 8, 10, 11, 15, 16, 18, 19, 21, 26–32, 46, 47].

As presented in Figs. 3 and 4, symptoms typically develop at about 6.4 years post-implantation, and explantation occurs at an average of 12.3 years. This suggests patients typically live with symptoms for approximately 6 years before having their implants removed.

As can be seen in Fig. 5, across the combined cohort of 1073 patients who underwent explantation for systemic symptoms, 879 patients (81.9%) reported subsequent symptom improvement. This aggregate finding suggests that across all studies, approximately four out of five patients experienced symptom improvement following explantation. As shown in Fig. 9, the average percentage reduction in symptoms following explantation is 55.1% or 6 symptoms.

The meta-analysis in Fig. 10 provides evidence supporting symptom improvement following explantation while acknowledging the variable magnitude of improvement that patients might experience.

However, Newby et al*.* highlighted that despite experiencing some health improvements, women who underwent explantation continued to have more severe physical symptoms and poorer mental health compared to those without BII [9].

### Silicone Adjuvant Theory

The silicone adjuvant theory proposes that silicone may act as an immune-stimulating agent, increasing risk of autoimmune and rheumatic disorders [33, 48], with BII being an autoimmune or inflammatory reaction triggered by exposure, manifesting as a spectrum of symptoms resembling connective tissue disease [8]. The predominance of silicone implants among the reported cases (70%) aligns with this theory.

Lee et al*.* argued that the presence of systemic disease in patients with saline implants challenges this theory [1]. However, Wee et al*.* explained that silicone is present in saline implant shells, possibly explaining similar symptom relief after explantation of both saline and silicone implants [21].

Research found that patients with silicone implants exhibited a high T4/T8 ratio [23] and immune activation through TH1/TH17 cells, promoting the production of pro-inflammatory cytokines [49], that perpetuate the inflammatory reaction, which locally causes capsular contracture and systemically may trigger autoimmune diseases [36, 50]. Three serum cytokines (IL-22, IL-13, IL-17A) were found to be significantly higher in BII patients [25].

Further immune markers were explored by Khan et al., who reported elevated oxylipins in symptomatic breast implant cohorts, correlating with symptom severity and foreign body response [22].

Silicone implants have been linked to autoantibody production, which normalize following explantation [23, 51]. Several studies have reported a high prevalence of antinuclear antibodies (ANA) in individuals with silicone breast implants [15, 25, 45, 46]. Shoaib et al*.* found evidence of abnormal immune activation in spinal fluid samples, reinforcing the hypothesis of diffuse immune involvement [34]. In this systematic review, the pooled prevalence of ANA positivity among BII patients was 24% (95% CI: 21–27%) compared to the general population which is 15% [52], suggesting a noteworthy prevalence of this autoimmune marker in almost a quarter of affected individuals, potentially indicating an immunological component to the condition.

Certain individuals may be predisposed to an immune response against implant material [16], with identified genetic HLA predispositions (e.g., HLA-DR5, HLA-DQ2, HLA DRB1 and HLA DQB1) as risk factors for autoimmune-like reactions following silicone exposure [14, 53, 54].

The overall rupture rate of 28.5% found in this review indicates that more than one in four BII patients experienced implant rupture or bleed. This substantial proportion suggests that implant integrity issues may play a significant role in the development of BII symptoms for many patients [19]. Moreover, implant rupture has been correlated with symptom severity [37]. Evidence of silicone migration was found in lymph nodes, lungs, and connective tissues and was linked to chronic inflammatory infiltrates [47], that ultimately evolved into systemic symptoms [36, 55].

Furthermore, inflammation of the capsule surrounding implants (giant cell reaction, foreign body granulomas, lymphocytic infiltrates, macrophages or perivascular inflammation) has been widely reported with a significant association to BII [1, 18]. As seen in Fig. 15, the overall mean prevalence in this review was 58.4%, with the clustering of three studies [1, 24, 34] at higher prevalence rates, which suggests that capsular inflammation is indeed common in these patients. Glicksman et al*.* emphasized that synovial metaplasia and epithelioid histiocytic reactions are markers of the immune system’s response to foreign bodies [25].

About capsular contracture, the overall rate was 40.6%, leading to significant physical symptoms such as breast pain, muscle aches, and difficulty breathing due to chest wall restriction [21]. It may result from an inflammatory reaction [21] and it has been associated with systemic symptoms and circulating immune complexes [56].

The predominance of smooth-surfaced implants, although less associated with BIA-ALCL (Breast Implant-Associated Anaplastic Large Cell Lymphoma) than textured ones [57], are more prone to capsular contracture, which itself has been linked to local inflammation and potentially to BII-like symptoms [58, 59]. In addition, some experimental data suggest that smooth implants may be more susceptible to microbleeding and subsequent inflammatory responses, due to their higher mechanical mobility within the capsule [60].

Overall, the evidence suggests a strong link between silicone breast implants and immune activation, with a notable prevalence of autoimmune markers, inflammatory responses, implant rupture and contracture in BII patients.

### History of Autoimmune Diagnosis

Research found that women with BII were significantly more likely to have been diagnosed with autoimmune diseases compared to those without this condition [9, 10, 61]. In this review, approximately 20.7% of individuals with BII had a reported history of autoimmune diagnosis, with an overall mean prevalence of 28.8%.

The distribution pattern shown in Fig. 12 suggests that while BII patients may experience a wide spectrum of autoimmune manifestations, certain conditions—particularly Rheumatoid Arthritis and Sjögren’s syndrome are more common. This is aligned with the most common symptoms experienced by patients (Fig. 6) and might offer valuable clues about potential pathophysiological mechanisms connecting breast implants to autoimmune phenomena, potentially involving specific immune pathways or tissue vulnerabilities that should be explored in further research to determine whether the placement of breast implants in women with autoimmune diseases is safe or if it may exacerbate or trigger adverse immune responses, causing BII.

### Positive Microbial Culture

In this review, 193 out of 548 BII patients (35.22%) had positive microbial growth around the implants. Biofilm formation can contribute to a chronic inflammatory response, capsular contracture, other systemic symptoms and it can trigger autoimmune-like reactions [16].

Studies have identified a significantly higher rate of positive cultures in BII patients, with *C. acnes* being the predominant microorganism [1, 62]. This is consistent with the results shown in Fig. 14. This distribution pattern along with the predominance of gram-positive bacteria (91.03%), strongly suggests that the microbial profile associated with BII is dominated by normal skin flora, particularly lipophilic organisms adapted to colonize the implant microenvironment [61, 63, 64].

This raises important questions about whether these microorganisms play a causal role in BII pathogenesis or represent incidental colonization detected during explantation.

The substantial overrepresentation of *C. acnes* suggests this organism may deserve particular attention in future research examining potential microbial triggers or contributors to breast implant-associated symptoms.

Campbell et al*.* noted that bacterial infection development could be influenced by suppressed natural killer cell function in the presence of breast implants, which normalizes after explantation [51].

### Somatization Disorder

As shown in Fig. 11, depression and anxiety emerge as the most prominent psychiatric conditions prior to implantation in women with BII. This is aligned with Bresnick et al*.* noting that these are predictive factors for systemic symptoms, exacerbated by environmental stressors such as the Internet and social media [20].

Research indicates a significant correlation between breast implants and various mental health diagnosis, with elevated psychological distress, anxiety, depression [9, 10, 19, 25, 31, 39, 65] and neurotic personality traits [3, 66] playing a significant role in symptom development. Approximately 16.5% of individuals with BII (338 out of 2045 patients) had a reported history of mental illness, with an overall mean prevalance of 26.82%, in this review.

Social media plays a crucial role in amplifying these concerns, reinforcing shared experiences that could contribute to symptom manifestation [38, 67]. Research found that nearly all women with BII engaged with online support groups, BII webpages, and health-related websites at a higher rate than those without BII, increasing anxiety, symptom awareness and spreading misinformation [9, 10, 68, 69]. Valente et al*.* found that social network support groups influenced the decision to undergo explantation in 87.2% of patients [2].

The proposition that anxiety or depression and subsequent somatization may contribute to BII symptoms, with psychological amplification playing a more significant role than a direct biological reaction to implants, has been postulated by many authors [17, 65], and is aligned with the findings of this review.

Interestingly, Glicksman et al*.* observed that an increasing number of reported symptoms does not necessarily correlate with an underlying disease process. In fact, the likelihood of identifying a biomedical cause decreases as symptom reporting increases [39].

The high proportion of cosmetic reasoning for implantation (92.6%) among patients with BII warrants attention. Cosmetic augmentation patients are generally younger and healthier at baseline, which may lead to greater awareness and concern about systemic symptoms following implantation, possibly contributing to higher symptom reporting rates [70]. Moreover, psychological expectations from cosmetic surgery may influence perception and reporting of adverse outcomes [71]. Finally, these patientes have been reported to have a higher rate of psychotropic drug use and this was strongly associated with lower satisfaction following implantation [72].

### Fibromyalgia

There is a substantial overlap between symptoms of fibromyalgia and BII, raising the possibility that they may represent the same underlying condition [41, 42]. A high prevalence of fibromyalgia has been observed in women with silicone breast implants [25], acting as an independent predictor for developing symptoms [38]. These findings align with the meta-analysis in Fig. 13 which reports an elevated prevalence of fibromyalgia among BII patients (12%) compared to the general population (2–3%) [73], suggesting a potential association between the two conditions.

### BII as a Clinical Entity

In this systematic review, 81.9% of patients reported symptom improvement after explantation, with a significant average symptom reduction of 55.1%, supporting a genuine association between breast implants and systemic symptoms. The findings of elevated ANA positivity (24%), increased autoimmune diagnoses (20.7%) and capsular inflammation (58.4%) reinforce the hypothesis of a pathological process involving immune dysregulation and chronic inflammation. Therefore, it suggests that BII represents a valid, complex clinical manifestation requiring individualized evaluation, as it certainly represents a diagnosis of exclusion.

While BII is not yet universally acknowledged as a distinct medical entity, the FDA has formally included it in its black box warning, emphasizing the need for thorough informed consent and shared decision-making [74]. Each patient must be approached individually and thoroughly screened for autoimmune and psychiatric conditions [75]. The mandatory Patient Decision Checklist should be carefully reviewed and discussed [74, 76].

In patients with known autoimmune disease with systemic involvement or fibromyalgia, implantation may pose unacceptable risks [77]. In individuals with significant psychiatric history, the decision should be even more carefully considered, with strong emphasis on informed consent and realistic expectations [72, 74, 76]. Patients with depressive disorders are at higher risk of unfavorable psychological outcomes after surgery, including dissatisfaction with the surgical results and psychosomatic complaints, and should therefore be referred to a psychiatrist prior to surgery when signs of depressive disorders or risk factors are identified [78, 79]. Above all, each case must be managed individually, prioritizing patient safety and long-term outcomes [75].

For patients proceeding with implantation, strict adherence to evidence-based surgical protocols—such as the 14-Point Plan and proven pocket irrigation solutions like Betadine triple (50 cc 10% Povidone-iodine + 1 g cefazolin + 80 mg gentamicin in 500 cc saline)—should be universally adopted to reduce bacterial contamination, capsular contracture and implant- associated anaplastic large cell lymphoma [80–83]. As seen in this review, 35.22% of patients with BII had positive microbial growth around the implants with the formation of biofilms contributing to chronic inflammation and capsular contracture, a polymicrobial issue [81, 84]. Routine long-term surveillance with imaging, including MRI or ultrasound every 2–3 years, is essential to monitor for silent complications, such as rupture [76].

### Reasoning for Exclusion Criteria

We excluded transgender women and cisgender men from the final analysis based on several methodological and biological considerations. Transgender women and cisgender men differ significantly from cisgender women in terms of hormonal profiles [85], immune system responses [86], and breast tissue anatomy [87]. The use of hormone replacement therapy (HRT) in transgender women—typically involving long-term estrogen and anti-androgen treatment—can modulate immune function and inflammatory pathways [88, 89], potentially affecting both the manifestation and severity of breast implant illness (BII) symptoms. Including these subgroups could therefore introduce confounding factors not applicable to cisgender women, the primary population under investigation. Moreover, uniformity in hormonal exposure and immunological background is essential for the accurate interpretation of inflammatory and autoimmune responses [14], which are central to the hypothesized pathophysiology of BII.

Transgender individuals may also exhibit distinct health-seeking behaviors, psychosocial experiences, and comorbidities—including higher baseline rates of depression and anxiety—compared to cisgender women [90], which could skew the reporting and perception of systemic symptoms and mental health diagnoses.

Finally, previous high-quality systematic reviews on BII have focused exclusively on cisgender female cohorts, citing similar concerns regarding heterogeneity and confounding variables [91]. By aligning with these methodological precedents, our study ensures consistency and comparability with the existing body of literature.

Given the significant safety concerns regarding Poly Implant Prothèse (PIP) implants, studies involving these devices were excluded from our systematic review. PIP implants were manufactured using industrial-grade silicone not approved for medical use, which led to substantially higher rates of rupture and silicone leakage compared to standard medical-grade implants [92]. Including studies with these implants would have introduced bias due to the manufacturing context of these devices, potentially confounding the relationship between silicone exposure and BII symptoms [93]. Consequently, excluding PIP implants was essential to preserve the methodological rigor and focus on medically approved implant types relevant to current clinical practice.

### Limitations

This review’s findings were subject to several limitations. The wide variety of study designs makes direct comparisons challenging and may contribute to inconsistent findings across studies.

The predominance of retrospective designs indicates that much of our knowledge comes from analyses of existing patient records rather than prospectively designed investigations, which may introduce selection bias since relying on medical records or self-reported data from individuals who have already experienced symptoms leads to a sample that may not represent the broader population of implant recipients. Also, recall bias may be present because patients reporting symptoms after implant placement may unintentionally misremember or emphasize certain details, especially if they strongly believe their implants caused their illness. Furthermore, observer bias may be a limitation since researchers analyzing past records may unconsciously interpret data in ways that align with existing hypotheses, particularly when diagnosing or classifying symptoms related to BII. Survivorship bias is also important in the sense that studies using patient records may underrepresent individuals who did not experience symptoms or did not seek medical attention, leading to an incomplete picture of the condition. Finally, information and confounding biases may be present since inconsistent or incomplete documentation in medical records can affect the accuracy of findings and other health conditions, lifestyle factors, or pre-existing conditions may contribute to symptoms.

The relatively short follow-up duration in most studies limits our understanding of the long-term trajectories of BII, including potential resolution patterns and late-emerging complications. Not all studies consistently report key variables such as implant characteristics and patient demographics, thus a substantial proportion of implant characteristics were categorized as 'unknown' in our review, as illustrated in Fig. 2. Rather than exclude these cases, which would have significantly reduced the study population and potentially introduced selection bias, we included them to transparently represent the limitations of the available evidence.

The concentration of studies in recent years suggests that our understanding of BII is still evolving, with more comprehensive and methodologically rigorous studies emerging as awareness of the condition increases. These findings underscore the need for standardized research approaches to BII, including consistent outcome measures, longer follow-up periods, and comprehensive reporting of implant characteristics and patient demographics. Such standardization would facilitate more robust meta-analyses and improve our understanding of risk factors, disease mechanisms, and optimal management strategies for this challenging condition.

## Conclusion

Breast Implant Illness (BII) presents as a multifaceted condition with a broad spectrum of systemic symptoms, predominantly musculoskeletal and constitutional in nature. The evidence suggests a strong association between breast implants and inflammatory, autoimmune and microbial responses. Psychological factors also play a crucial role in the BII experience, with high rates of depression, anxiety, and somatization tendencies observed among affected individuals.

This review supports BII as a real, multifactorial clinical entity involving immune dysregulation, chronic inflammation, and microbial biofilms. These findings underscore the importance of individualized assessment, screening for autoimmune and psychiatric conditions, informed consent and adherence to surgical protocols such as the 14-Point Plan and antimicrobial irrigation to reduce complications.

## Supplementary Information

Below is the link to the electronic supplementary material.

Supplementary file1 (DOCX 15 kb)

Supplementary file2 (DOCX 19 kb)
