# Supplementary material for: Breast Implant Illness: Symptoms, Outcomes with Explantation and Potential Etiologies—A Systematic Review and Meta-analysis
Source: Aesthetic Plast Surg. 2025 Aug 11;49(23):6600–20. doi: 10.1007/s00266-025-05142-x (PMC12738613; doi:10.1007/s00266-025-05142-x)
Supplement: Supplementary file 1 — Supplementary file1 (DOCX 15 kb) [file 266_2025_5142_MOESM1_ESM.docx]

| **Supplemental Table 1 - Risk of Bias - National Institute of Health** | |
| --- | --- |
| **Paper** | **Risk of bias** |
| Magno-Padron *et al.* [4] | Fair (6) |
| Amber Spit *et al.* [14] | Good (13) |
| Logothesis *et al.* [33] | Fair (6) |
| Maijers *et al.* [27] | Fair (9) |
| Azahaf *et al.* [30] | Good (12) |
| Solomon *et al.* [47] | Fair (8) |
| Katsnelson *et al.* [18] | Fair (5) |
| Miseré *et al.* [20] | Fair (5) |
| Miranda *et al.* [5] | Fair (7) |
| Wee *et al.* [23] | Fair (8) |
| Glicksman *et al.* [11,16,59,63] | Good (13) |
| Bird *et al.* [32] | Good (12) |
| Messa *et al.* [31] | Good (11) |
| Fryzek *et al.* [65] | Fair (10) |
| Berner *et al.* [66] | Good (11) |
| Metzinger *et al.* [15] | Good (11) |
| Serena *et al.* [35] | Good (12) |
| Lee *et al.* [1] | Good (11) |
| Bascone *et al.* [34] | Good (11) |
| Khan *et al.* [24] | Good (9) |
| Newby *et al.* [9] | Fair (9) |
| Shoaib *et al.* [43] | Good (12) |
| McGuire *et al.* [10] | Fair (6) |
| Colaris *et al.* [55] | Good (11) |
| Giltay *et al.* [64] | Fair (10) |
| Berben *et al.* [3] | Fair (6) |
| Bresnick *et al.* [21] | Good (8) |
| Vermeulen *et al.* [51] | Fair (8) |
| Jong *et al.* [67] | Fair (9) |
| Vojdani *et al.* [25] | Fair (8) |
| Halpert *et al.* [44] | Good (11) |
| Nagy *et al.* [26] | Good (10) |
| Tervaert *et al.* [42] | Fair (6) |
