# Supplementary material for: Breast Implant Illness: Symptoms, Outcomes with Explantation and Potential Etiologies—A Systematic Review and Meta-analysis
Source: Aesthetic Plast Surg. 2025 Aug 11;49(23):6600–20. doi: 10.1007/s00266-025-05142-x (PMC12738613; doi:10.1007/s00266-025-05142-x)
Supplement: Supplementary file 2 — Supplementary file2 (DOCX 19 kb) [file 266_2025_5142_MOESM2_ESM.docx]

| **Supplemental Table 2 - Characteristics of the 36 papers included in this systematic review** | | | | | |
| --- | --- | --- | --- | --- | --- |
| **Reference** | **Year** | **Study Design** | **Number of  Patients** | **Mean Age ± SD or  (Range) (years)** | **Mean Follow-Up  (Range) (months)** |
| Magno-Padron *et al.* [4] | 2020 | Cross Sectional | 168 | NR | NR |
| Amber Spit *et al.* [14] | 2021 | Prospective  Cohort | 467 | 48 ± 12 | 39.6 (24.0-48.0) |
| Logothesis *et al.* [33] | 1994 | Cross Sectional | 55 | 44 (26-72) | NR |
| Maijers *et al.* [27] | 2013 | Retrospective  Cohort | 80 | 47 (22-78) | 7 (1-216) |
| Azahaf *et al.* [30] | 2024 | Prospective  Cohort | 353 | 48.0 ± 11.7 | 12 (5-36) |
| Solomon *et al.* [47] | 1994 | Retrospective  Cohort | 176 | 45 (24-72) | 10 |
| Katsnelson *et al.* [18] | 2021 | Retrospective  Case Series | 248 | 45 (22-72) | 6 |
| Miseré *et al.* [20] | 2022 | Retrospective  Case Series | 29 | 50.6 (29.0-68.0) | NR |
| Miranda *et al.* [5] | 2020 | Cross sectional | 100 | 43.56 ± 1.06 | 15 |
| Wee *et al.* [23] | 2020 | Retrospective  Pre-Post | 750 | 45.9 ± 10.8 | 24 |
| Glicksman *et al.* [11,16,59,63] | 2021 | Prospective  Cohort | 50 | 44.5 (30.0-65.0) | 12 |
| Bird *et al.* [32] | 2022 | Prospective  Cohort | 140 | 43.2 ± 10.6  (24.0-69.0) | 6.7 (2.9-22.0) |
| Messa *et al.* [31] | 2023 | Retrospective  Cohort | 131 | 48.3 (26.0-75.0) | 23 (6-96) |
| Fryzek *et al.*  [65] | 2000 | Retrospective  Cohort | 1369 | 44.3 ± 10.0 | NR |
| Berner *et al.*  [66] | 2001 | Cross Sectional | 32 | 53.0 ± 9.5 | NR |
| Metzinger *et al.* [15] | 2022 | Retrospective  Cohort | 200 | 45.5 (29.0-73.0) | 5 (3-46) |
| Serena *et al.* [35] | 2023 | Retrospective  Cohort | 199 | 47.44 | 2 |

| **Supplemental Table 2 - (continued)** | | | | | |
| --- | --- | --- | --- | --- | --- |
| Lee *et al.* [1] | 2020 | Prospective  Cohort | 50 | 42.0 ± 0.8 | 3 |
| Bascone *et al.* [34] | 2023 | Retrospective  Cohort | 20 | 44.9 ± 9.8 | 18 |
| Khan *et al.* [24] | 2024 | Case Control | 46 | 47.39 ± 9.73 | NR |
| Newby *et al.* [9] | 2021 | Cross Sectional | 165 | 42.2 ± 11.9  (20.0-76.0) | NR |
| Shoaib *et al.* [43] | 1996 | Prospective  Cohort | 26 | 39.2 (23.0-64.0) | NR |
| McGuire *et al.* [10] | 2024 | Retrospective  Case Series | 72 | 46.3 (28.0-62.0) | 7 (5-12) |
| Colaris *et al.* [55] | 2020 | Retrospective  Cohort | 85 | 52.7 (35.0-71.0) | NR |
| Giltay *et al.* [64] | 1993 | Retrospective  Cohort | 235 | 43 (19-73) | 78 (24-168) |
| Berben *et al.* [3] | 2022 | Cross Sectional | 201 | 46.2 ± 12.8 | NR |
| Bresnick *et al.* [21] | 2024 | Retrospective  Case Series | 60 | 47.5 (44.0-52.0) | NR |
| Vermeulen *et al.* [51] | 2003 | Cross Sectional | 319 | 54.0 ± 8.6 | NR |
| Tervaert *et al.* [42] | 2013 | Retrospective  Cohort | 32 | 49 (18-64) | NR |
| Jong *et al.* [67] | 2002 | Cross Sectional | 42 | NR | NR |
| Vojdani *et al.* [25] | 1992 | Case Control | 40 | 46 (28-64) | NR |
| Halpert *et al.* [44] | 2021 | Prospective  Cohort | 93 | 40.5 | NR |
| Nagy *et al.* [26] | 2023 | Case Control | 15 | 42 (31-62) | 9 |

**NR** not reported
